# Supplementary material for: Cytochalasins from the Ash Endophytic Fungus Nemania diffusa DSM 116299
Source: Molecules. 2025 Feb 19;30(4):957. doi: 10.3390/molecules30040957 (PMC11858462; doi:10.3390/molecules30040957)
Supplement: Supplementary file 1 [file molecules-30-00957-s001.zip › molecules-3412242-supplementary.pdf]

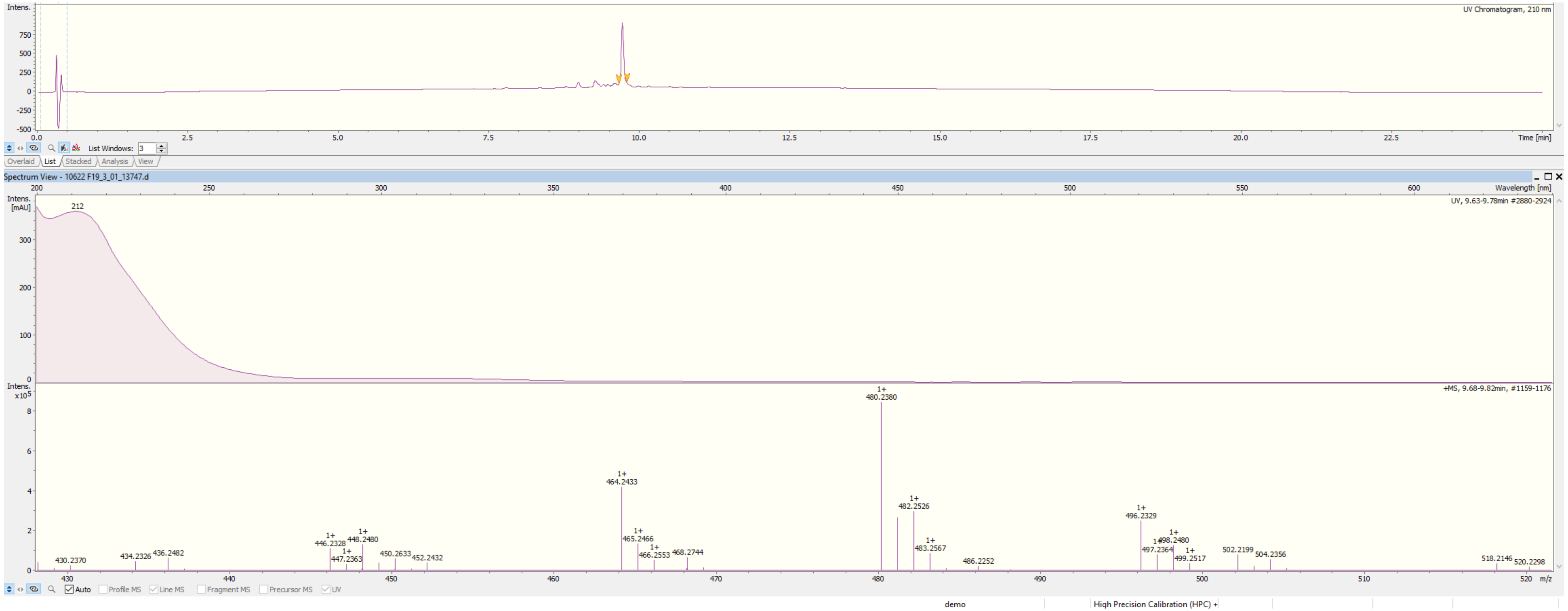

Figure S1. HR- ESI (+) MS data for new cytochalasin (**1**).

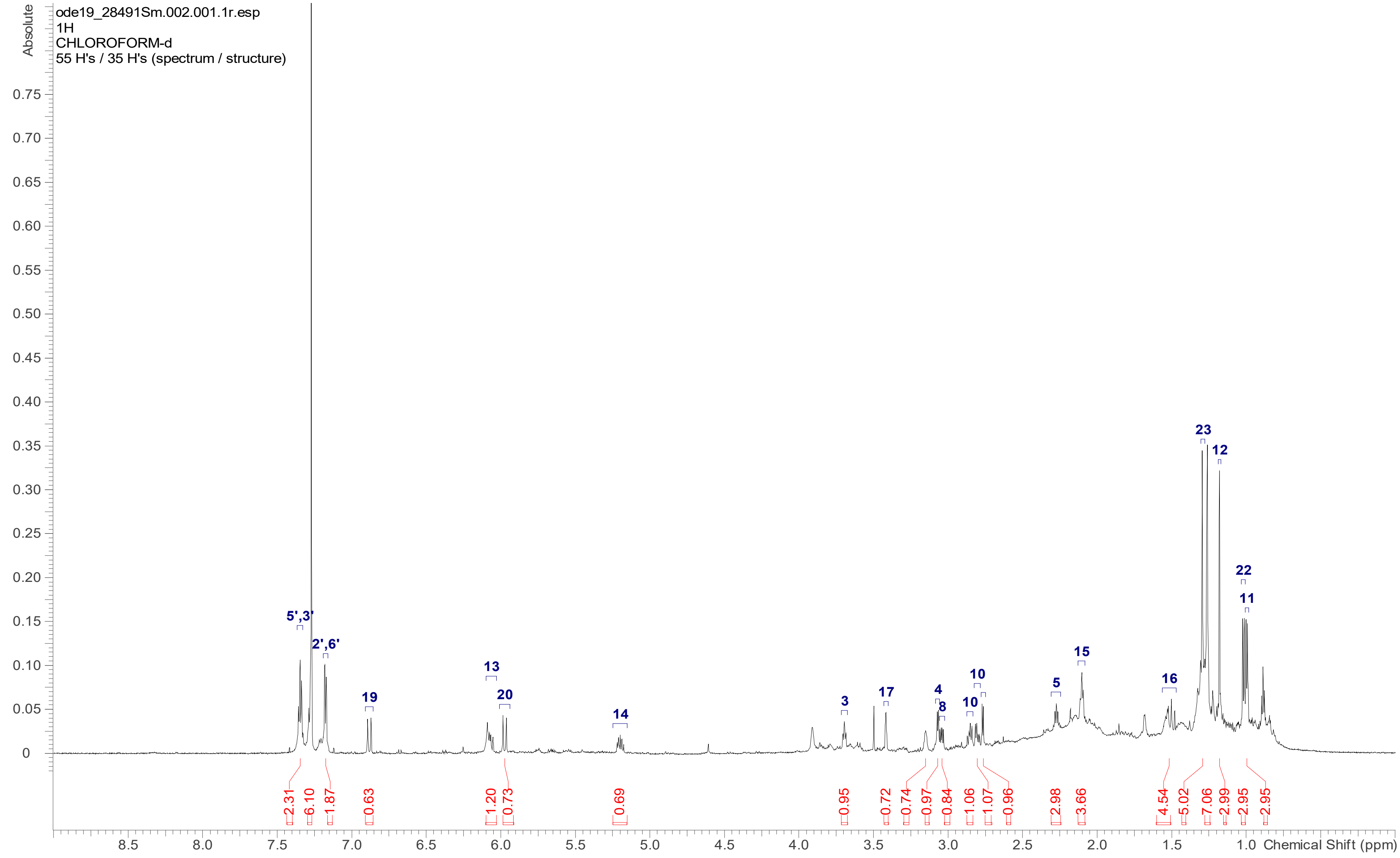

Figure S2. ^{1}H NMR spectrum (700 MHz, $CHCl_3$-*d*) of **1**.

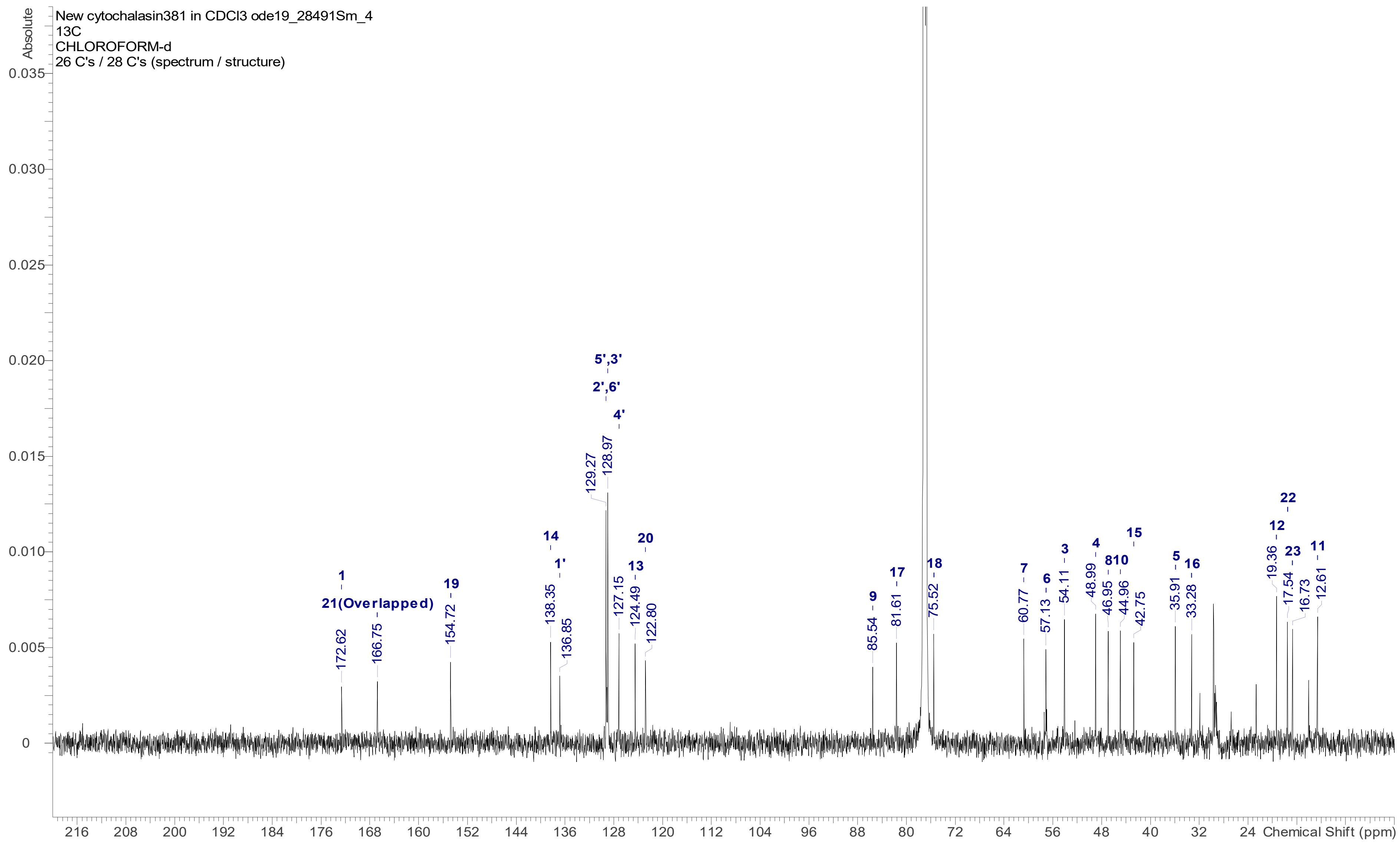

Figure S3. ^{13}C NMR spectrum (175 MHz, $CHCl_3$-*d*) of **1**.

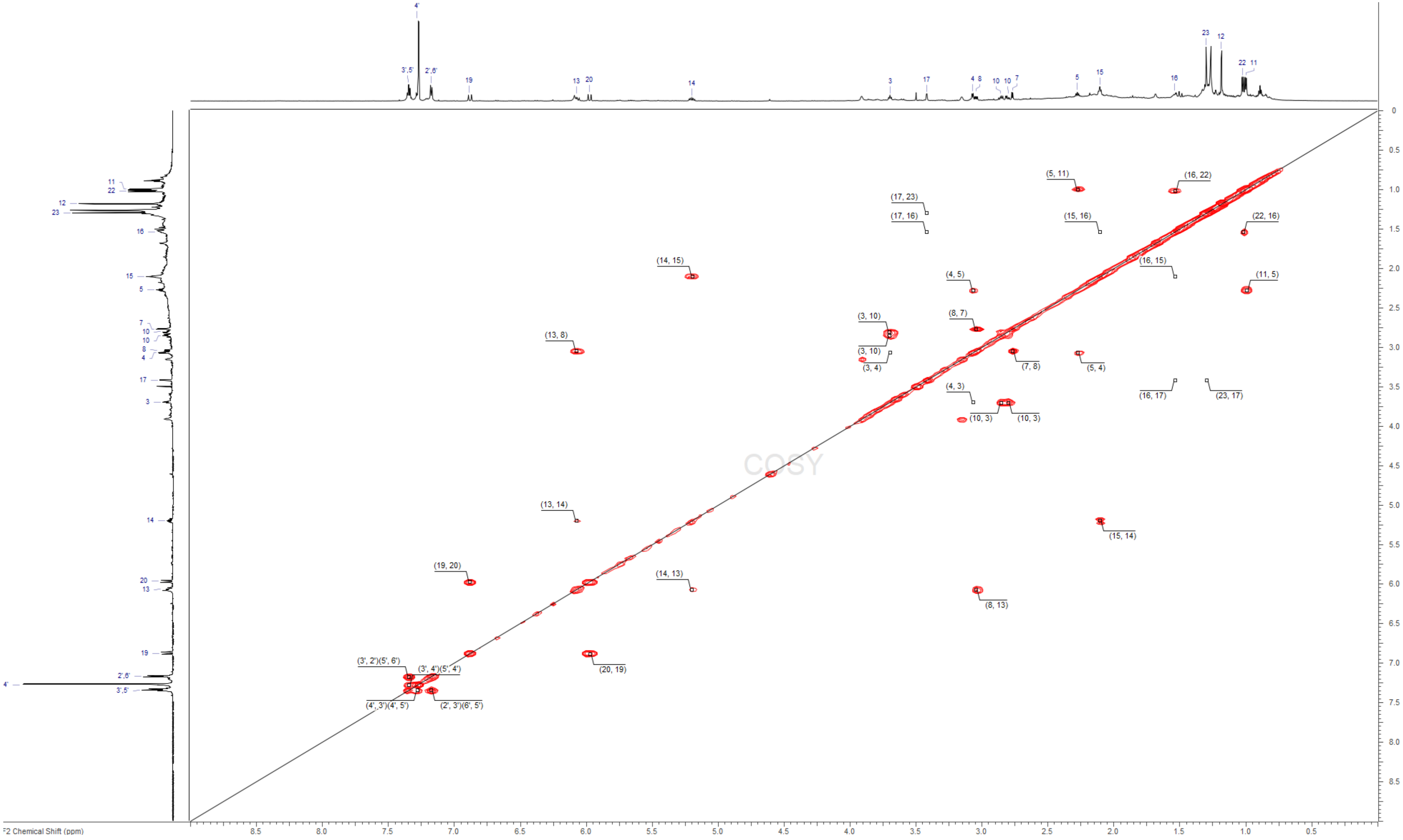

Figure S4. COSY NMR spectrum (700 MHz, $CHCl_3$-*d*) of **1**.

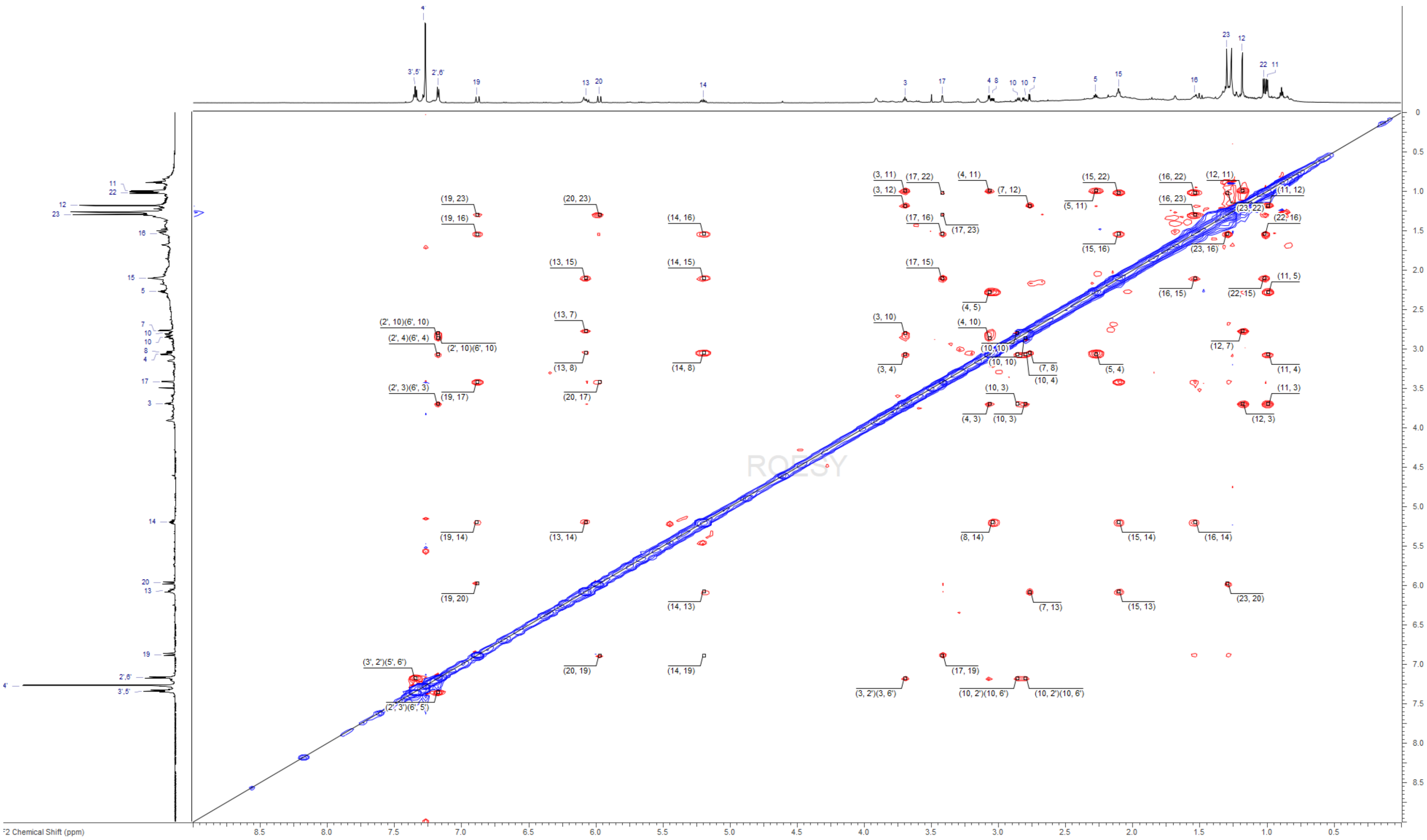

Figure S5. ROESY NMR spectrum (700 MHz, $CHCl_3$-*d*) of **1**.

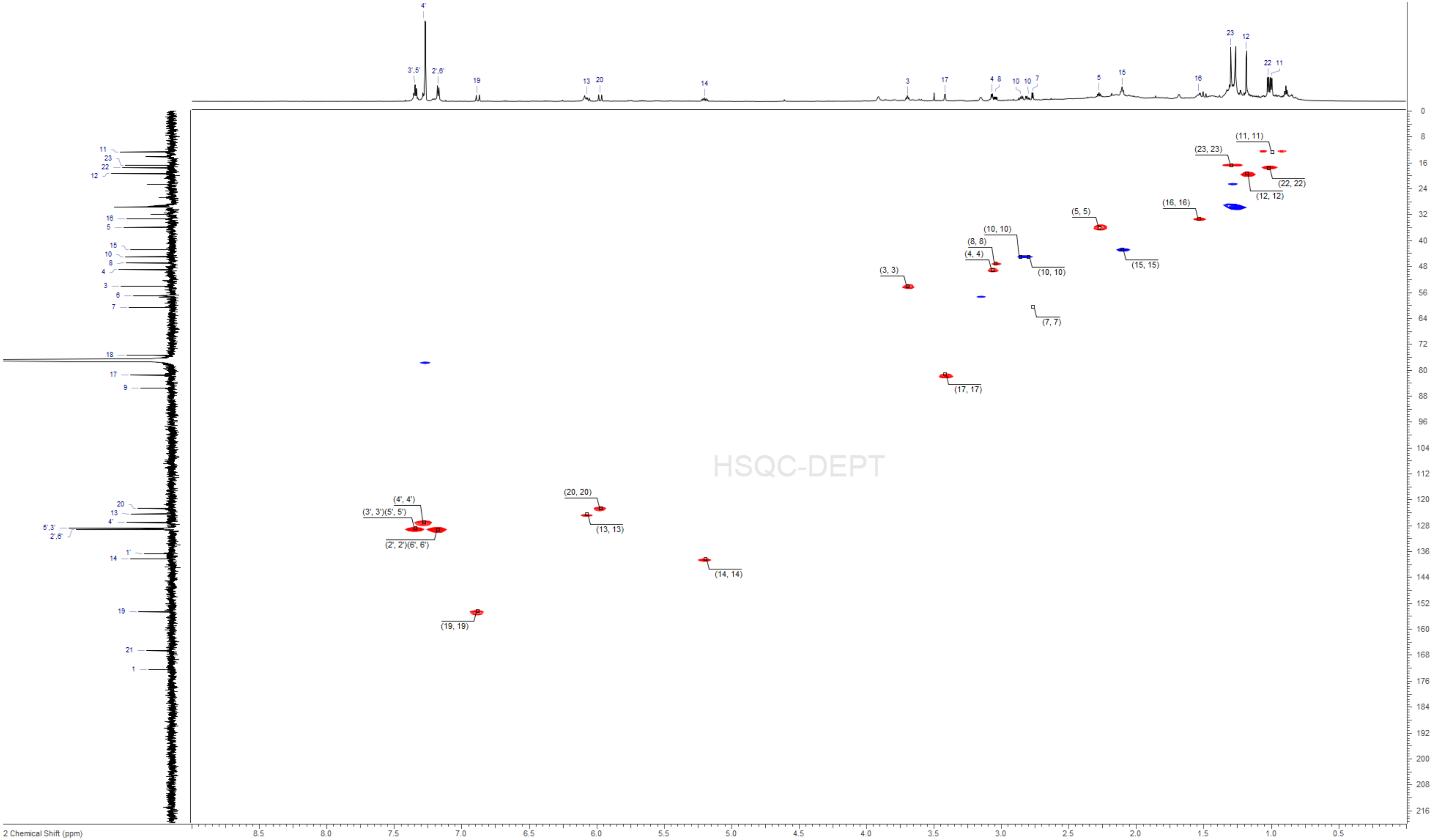

Figure S6. HSQC NMR spectrum (700 MHz, $CHCl_3$-*d*) of **1**.

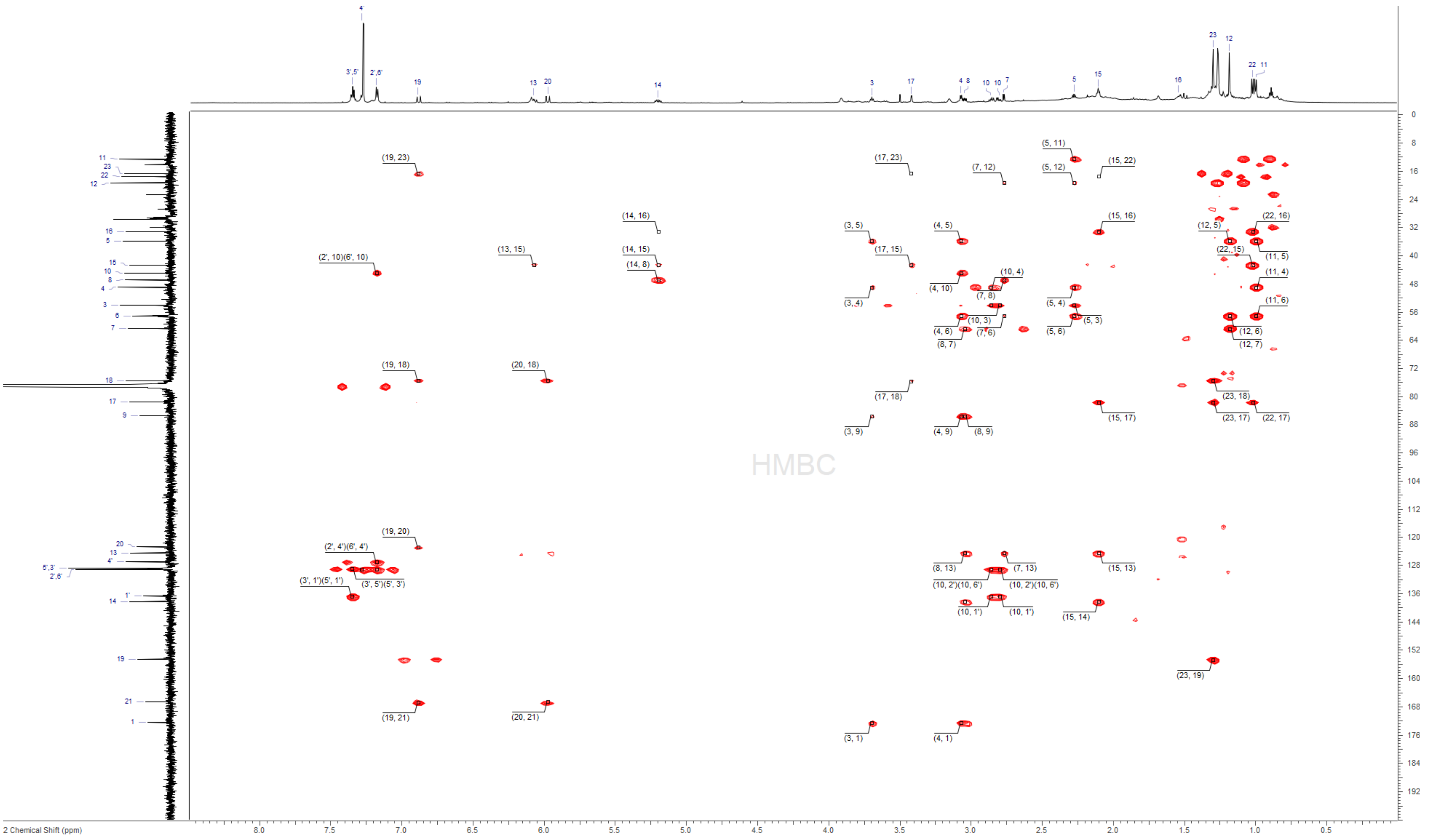

Figure S7. HMBC NMR spectrum (700 MHz, $CHCl_3$-*d*) of **1**.

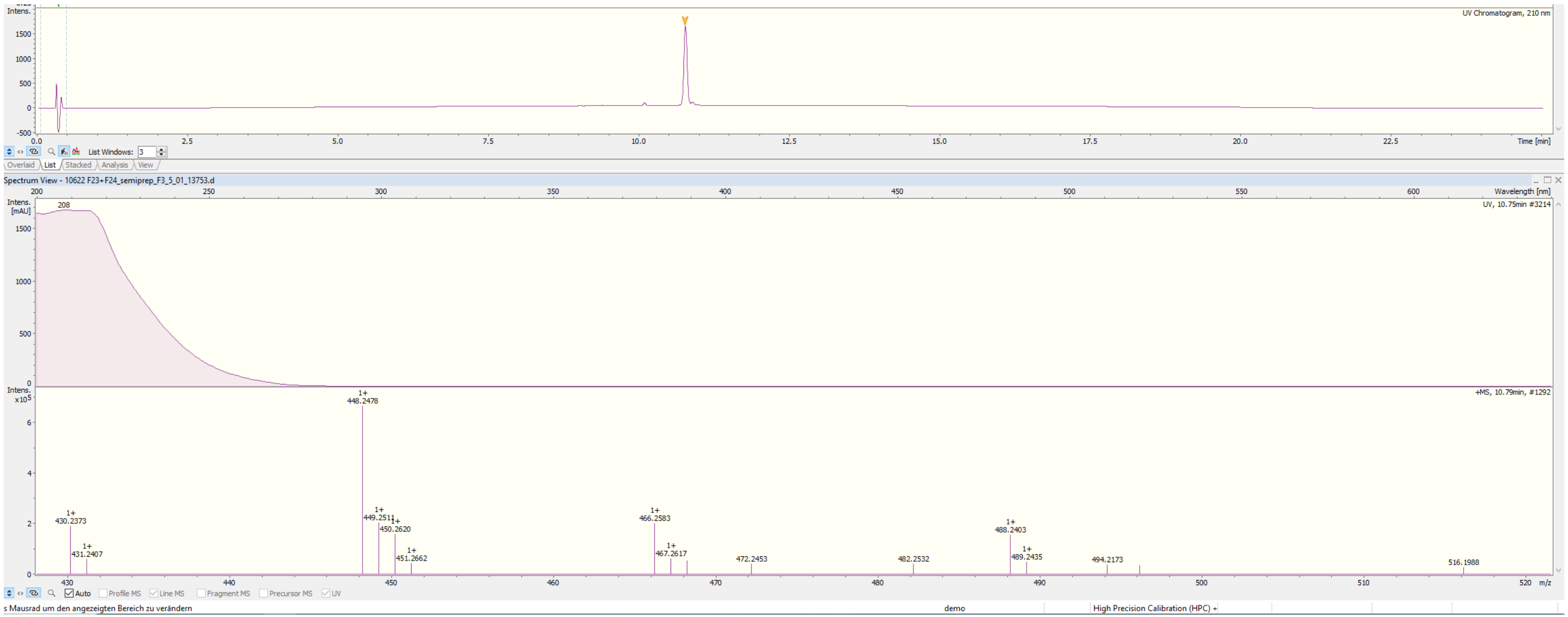

Figure S8: HR- ESI (+) MS data for (**2**).

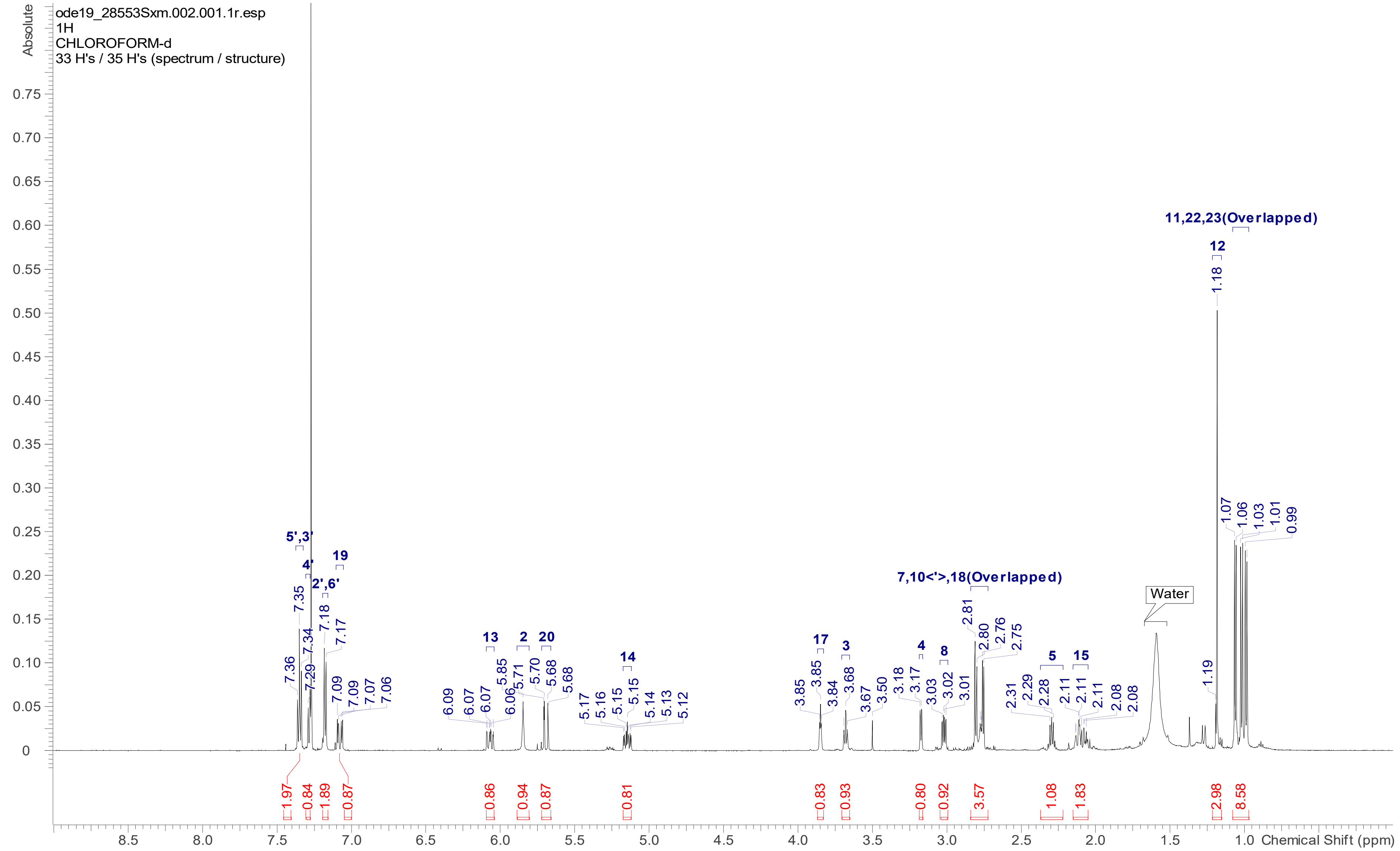

Figure S9. ^{1}H NMR spectrum (700 MHz, $CHCl_3$-*d*) of **2**.

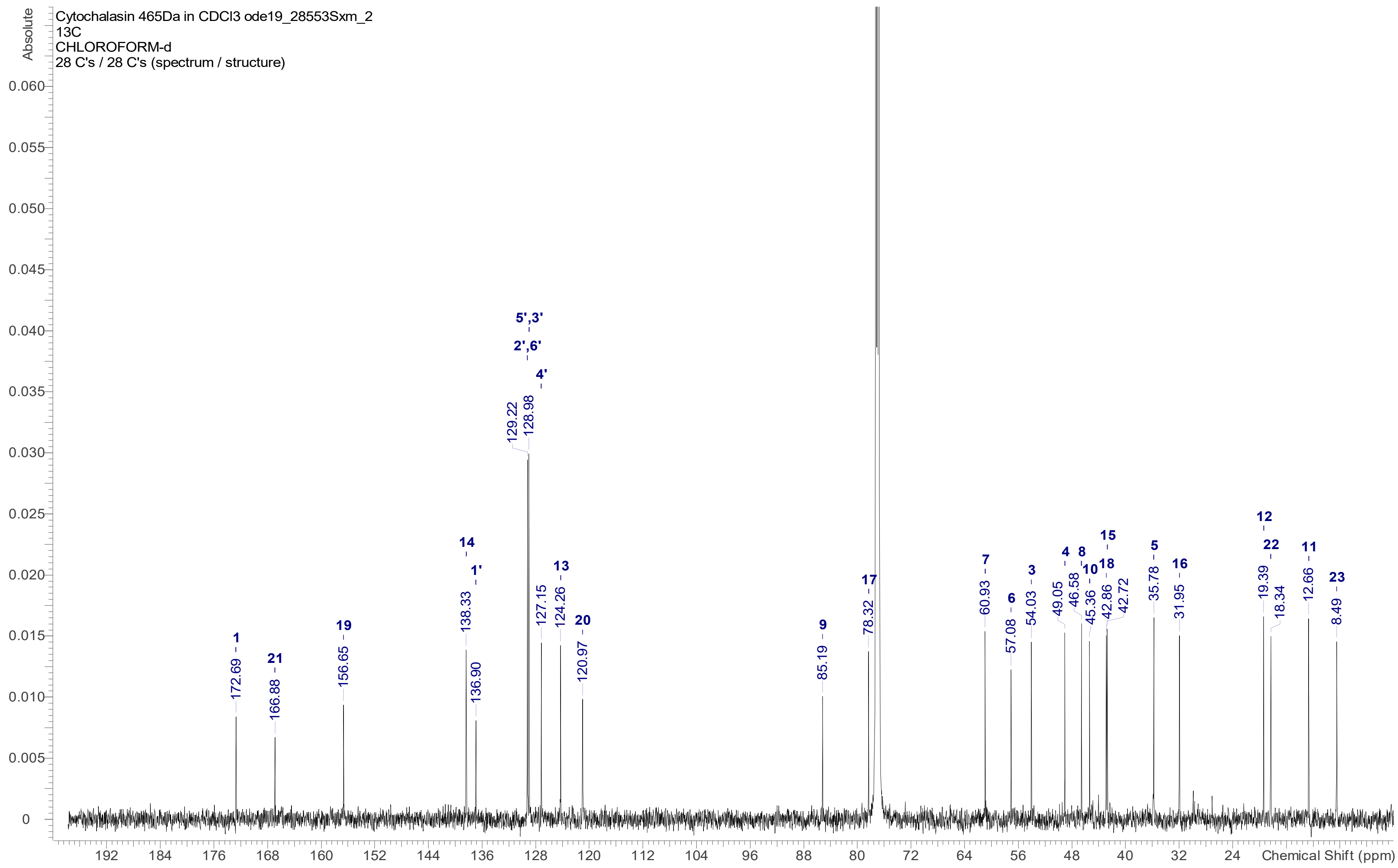

Figure S10. ^{13}C NMR spectrum (175 MHz, $CHCl_3$-*d*) of **2**.

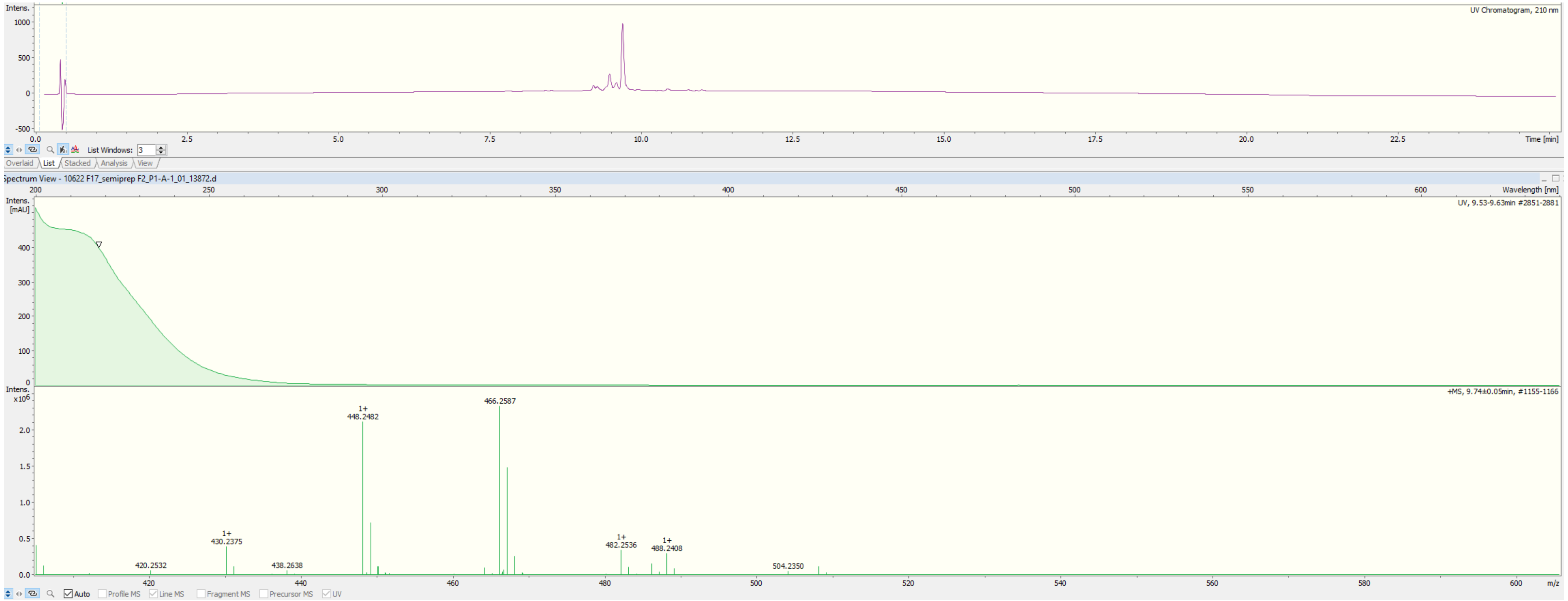

Figure S11: HR- ESI (+) MS data for cytochalasin Z_7 (**3**).

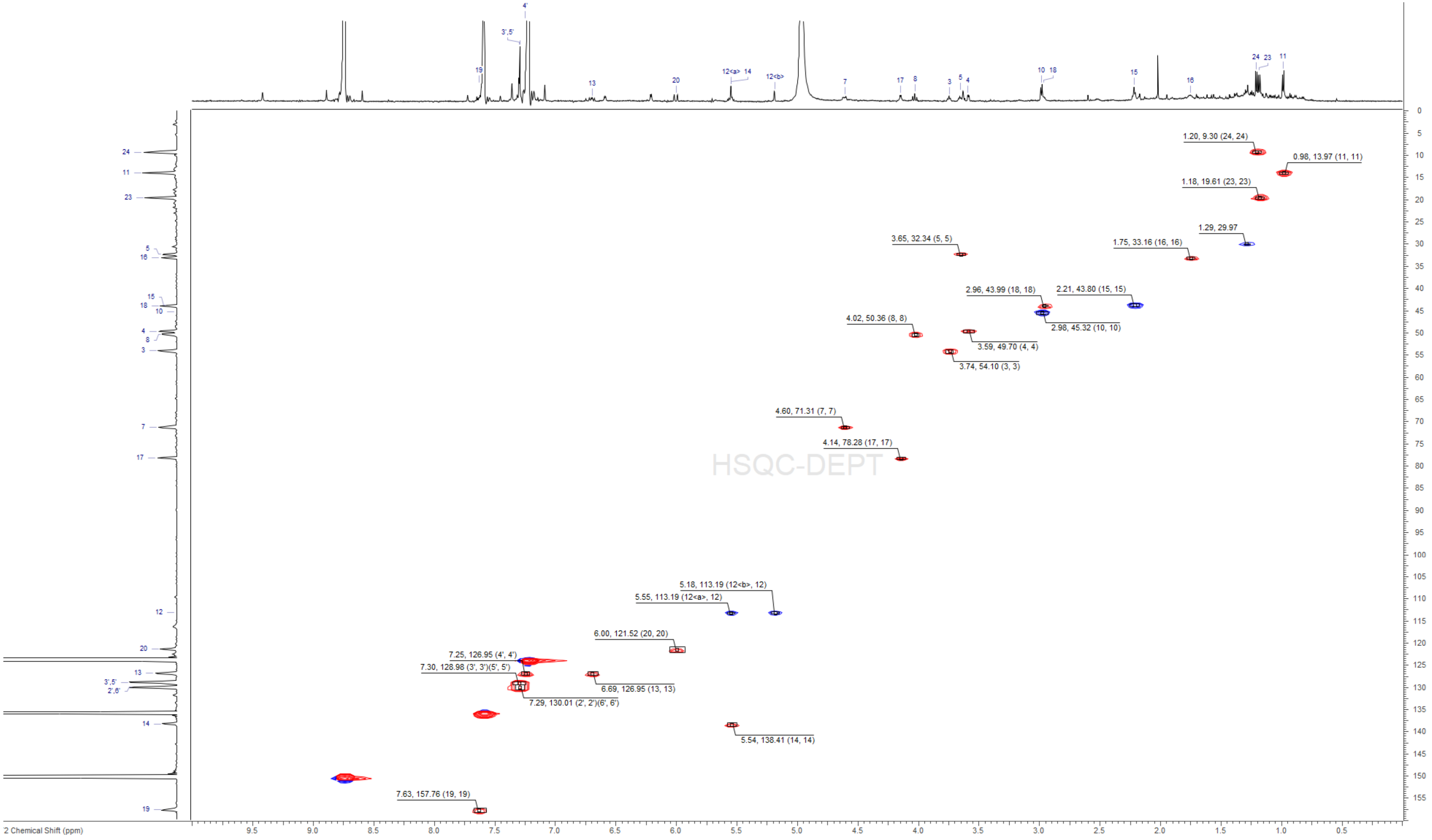

Figure S12. HSQC NMR spectrum (700 MHz, pyridin-d_5) of **3**.

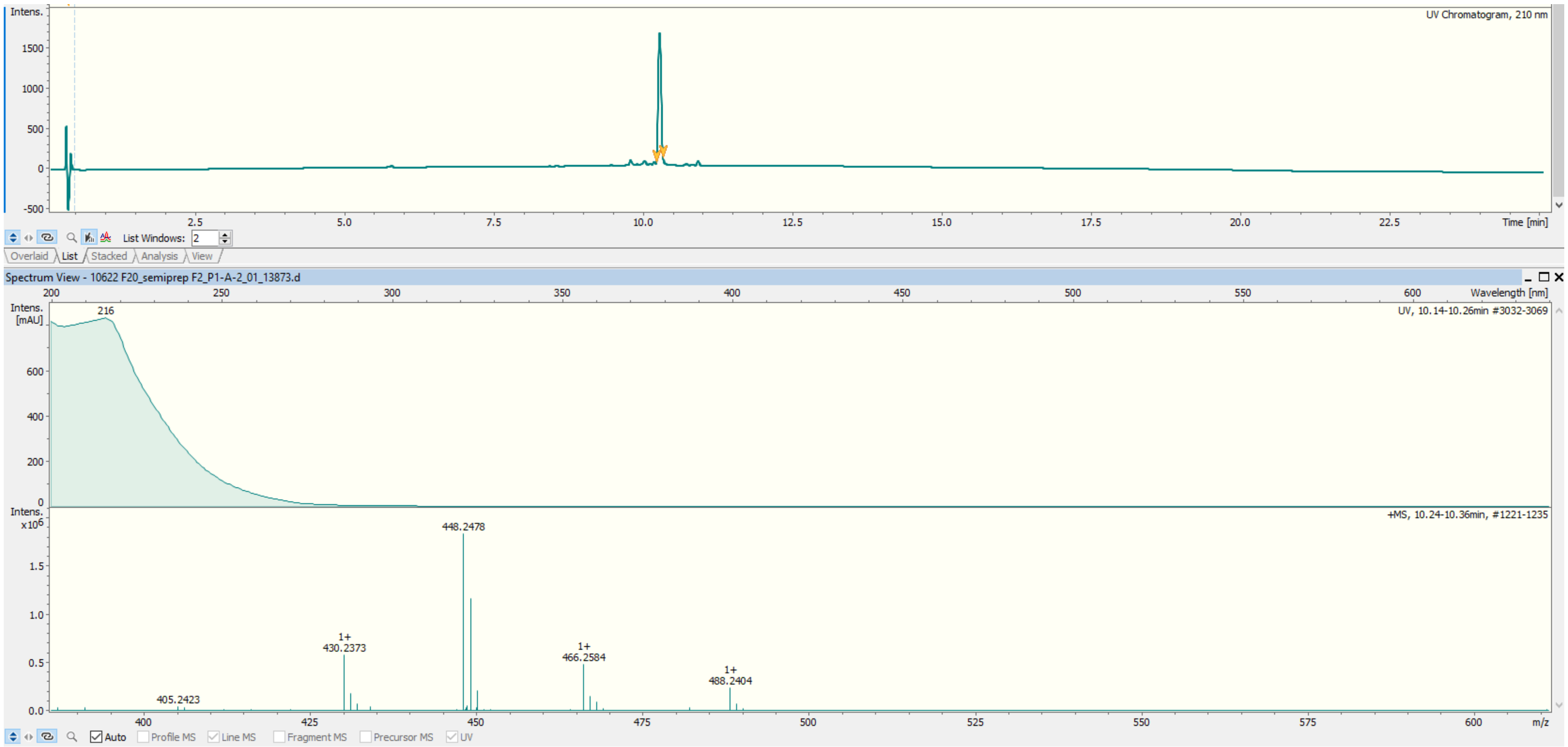

Figure S13: HR- ESI (+) MS data for cytochalasin Z_8 (**4**).

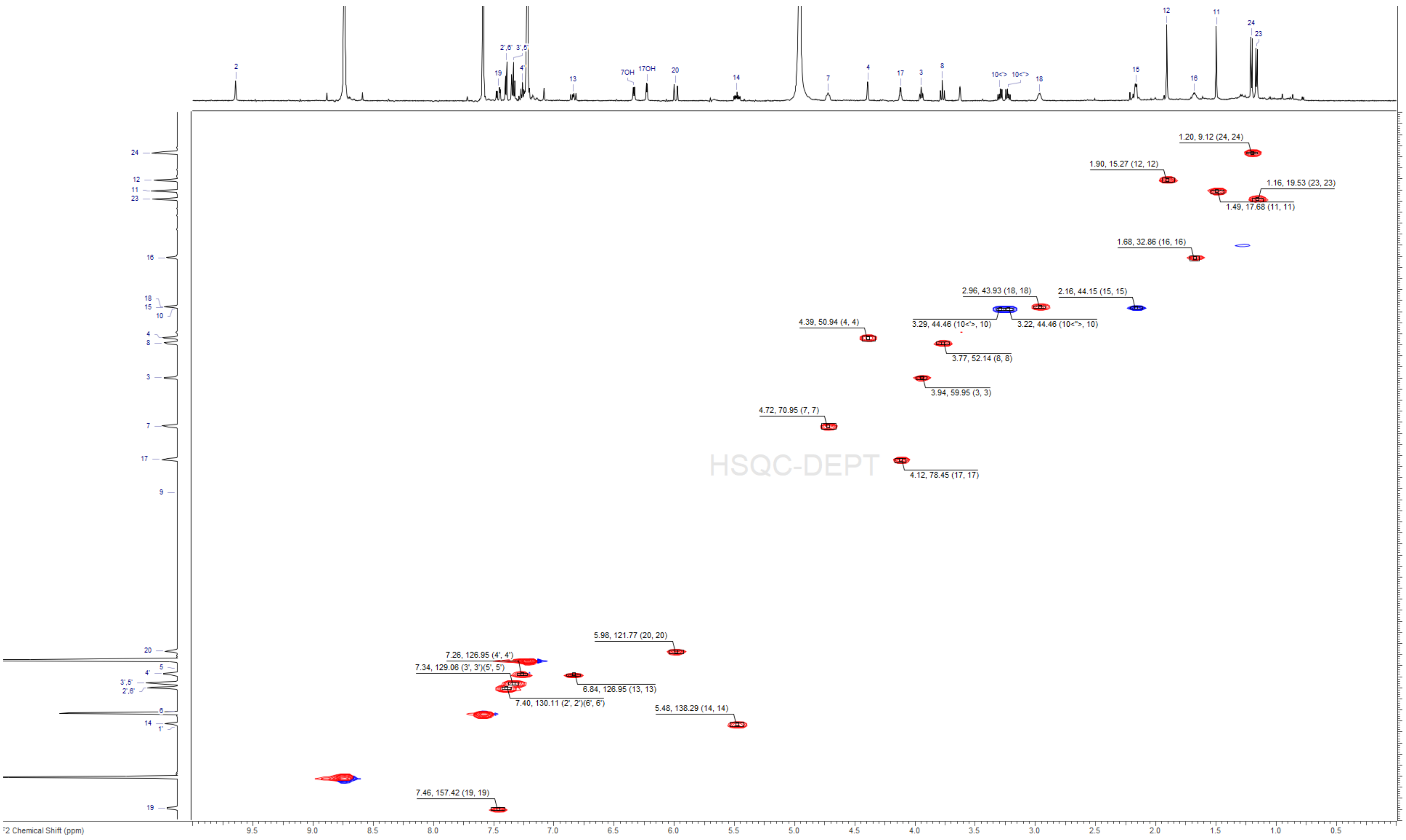

Figure S14. HSQC NMR spectrum (700 MHz, pyridin-d_5) of **4**.

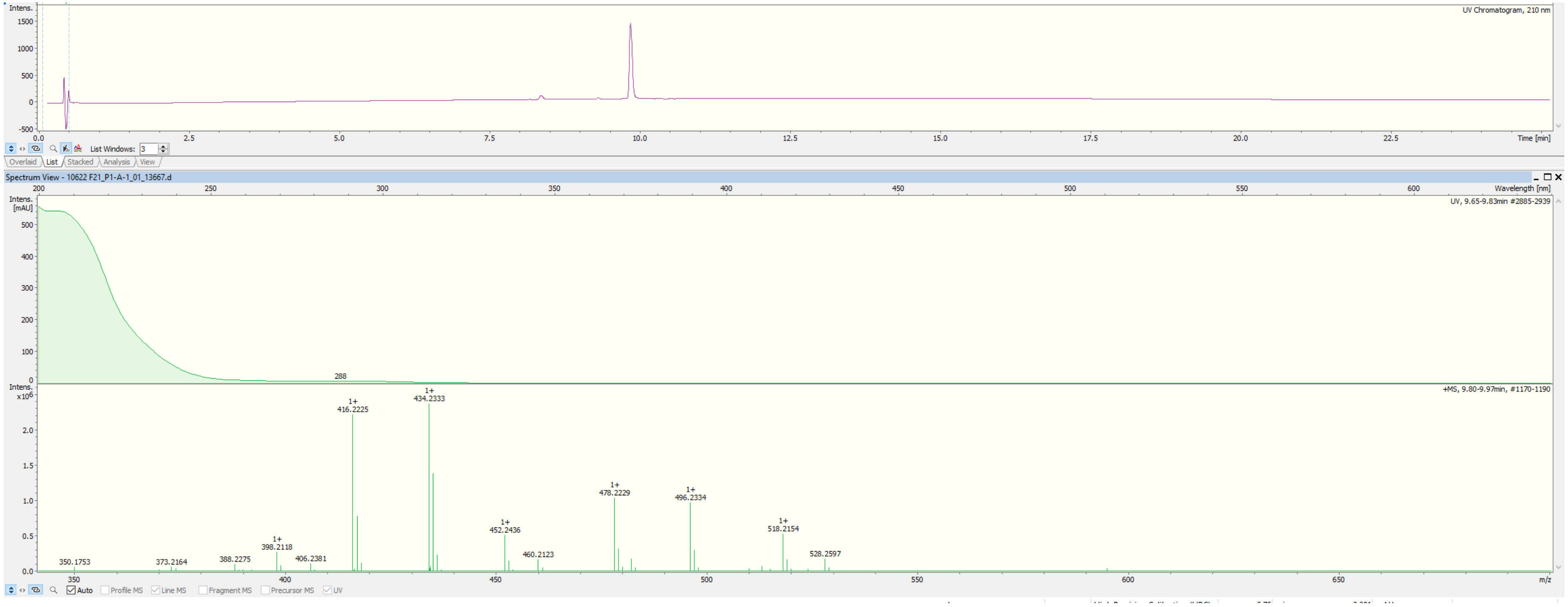

Figure S15: HR- ESI (+) MS data for Cytochalasin E (**5**).

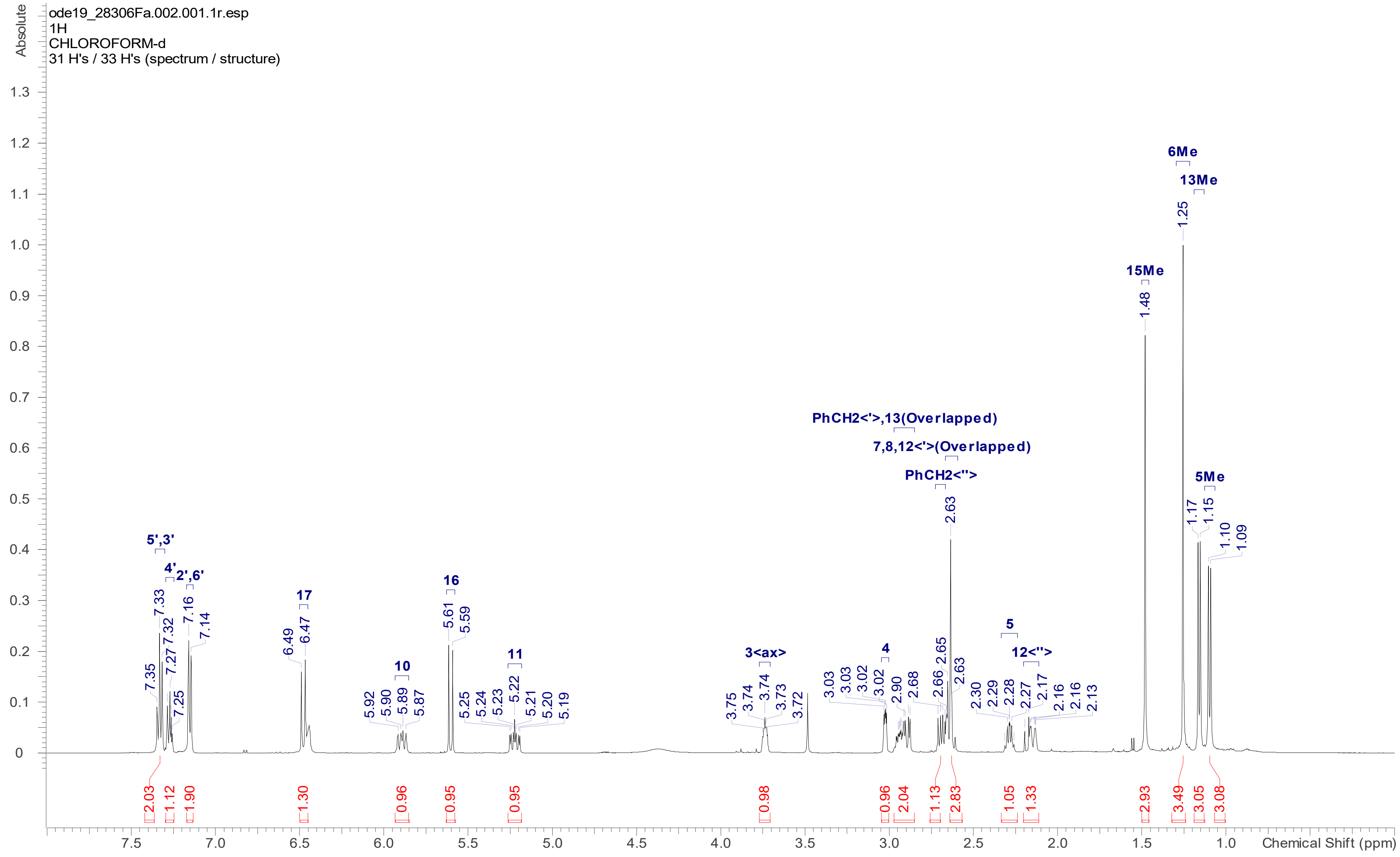

Figure S16. ^{1}H NMR spectrum (500 MHz, $CHCl_3$-*d*) of **5**.

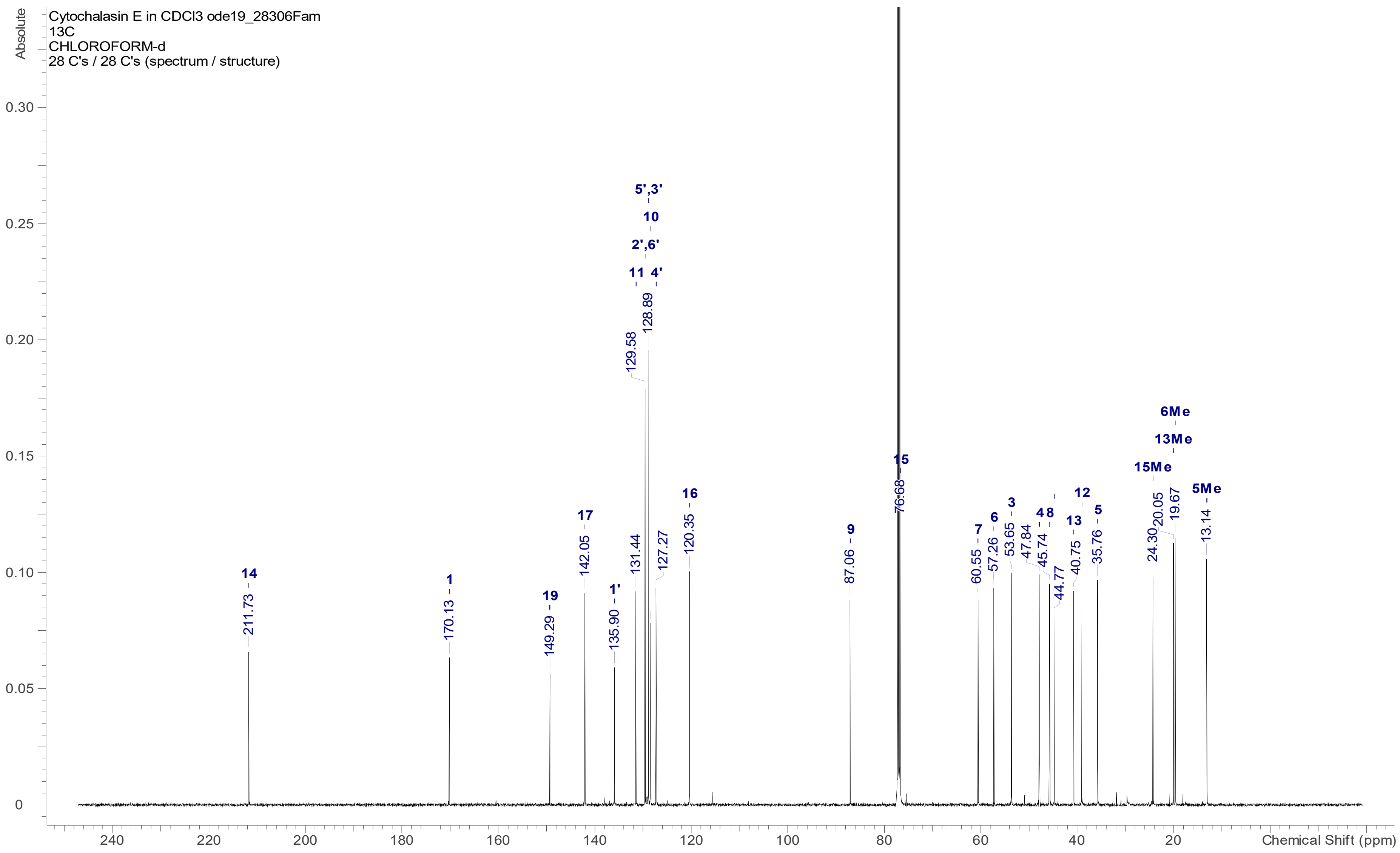

Figure S17. ^{13}C NMR spectrum (125 MHz, $CHCl_3$-*d*) of **5**.

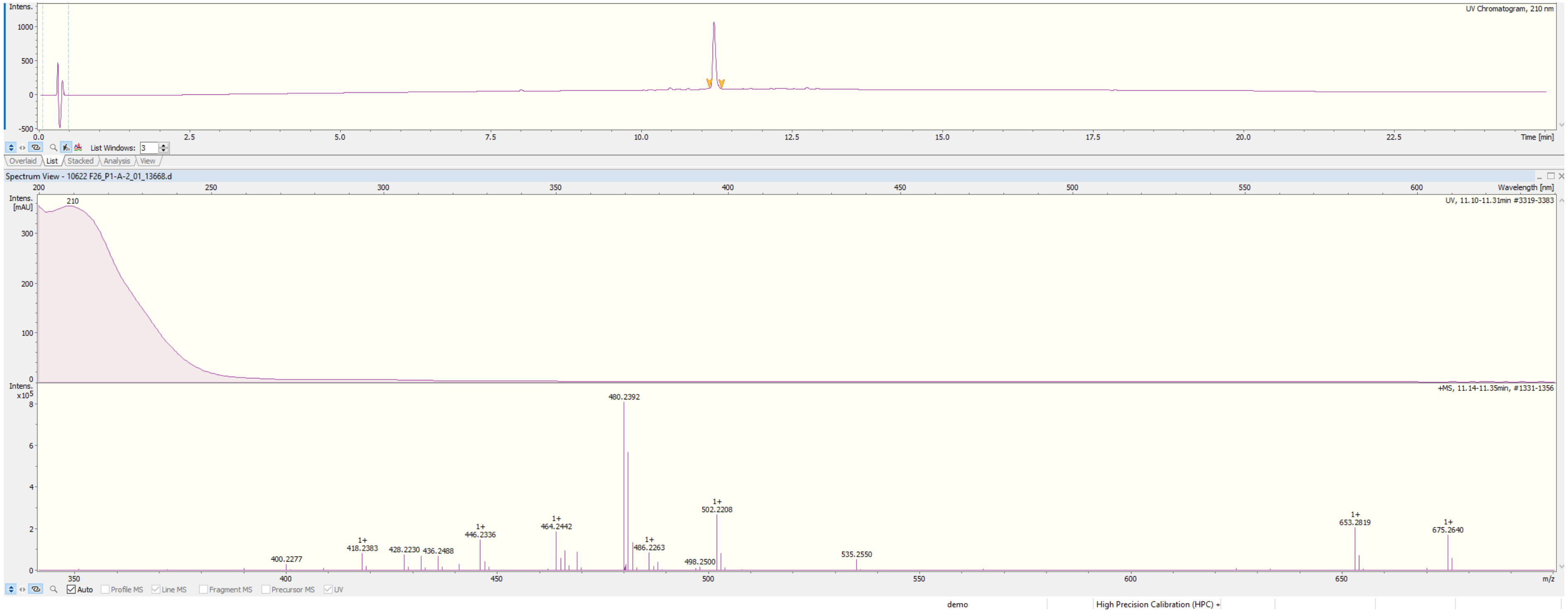

Figure S18: HR- ESI (+) MS data for **6.**

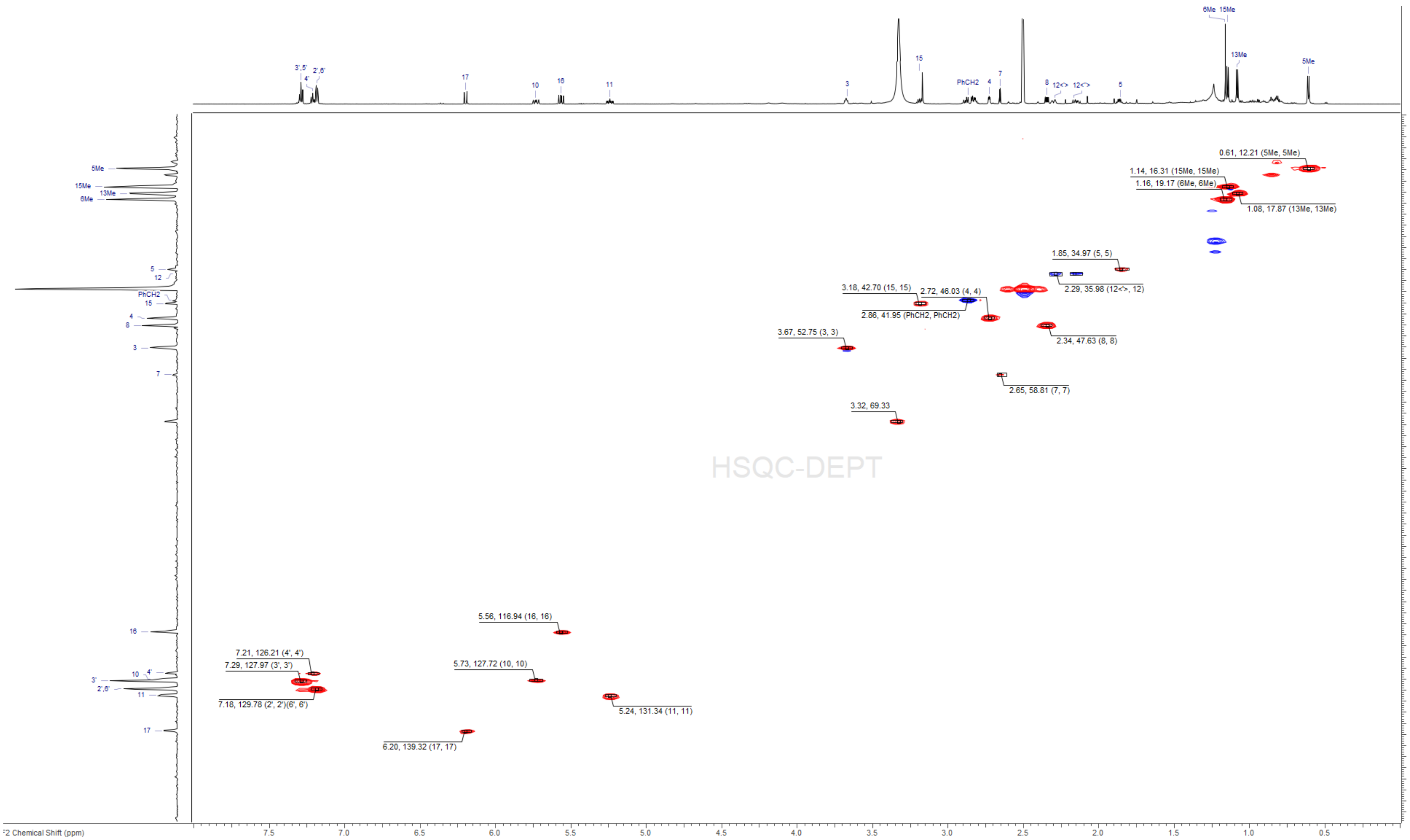

Figure S19. HSQC NMR spectrum (700 MHz, DMSO-d_6) of **6**.

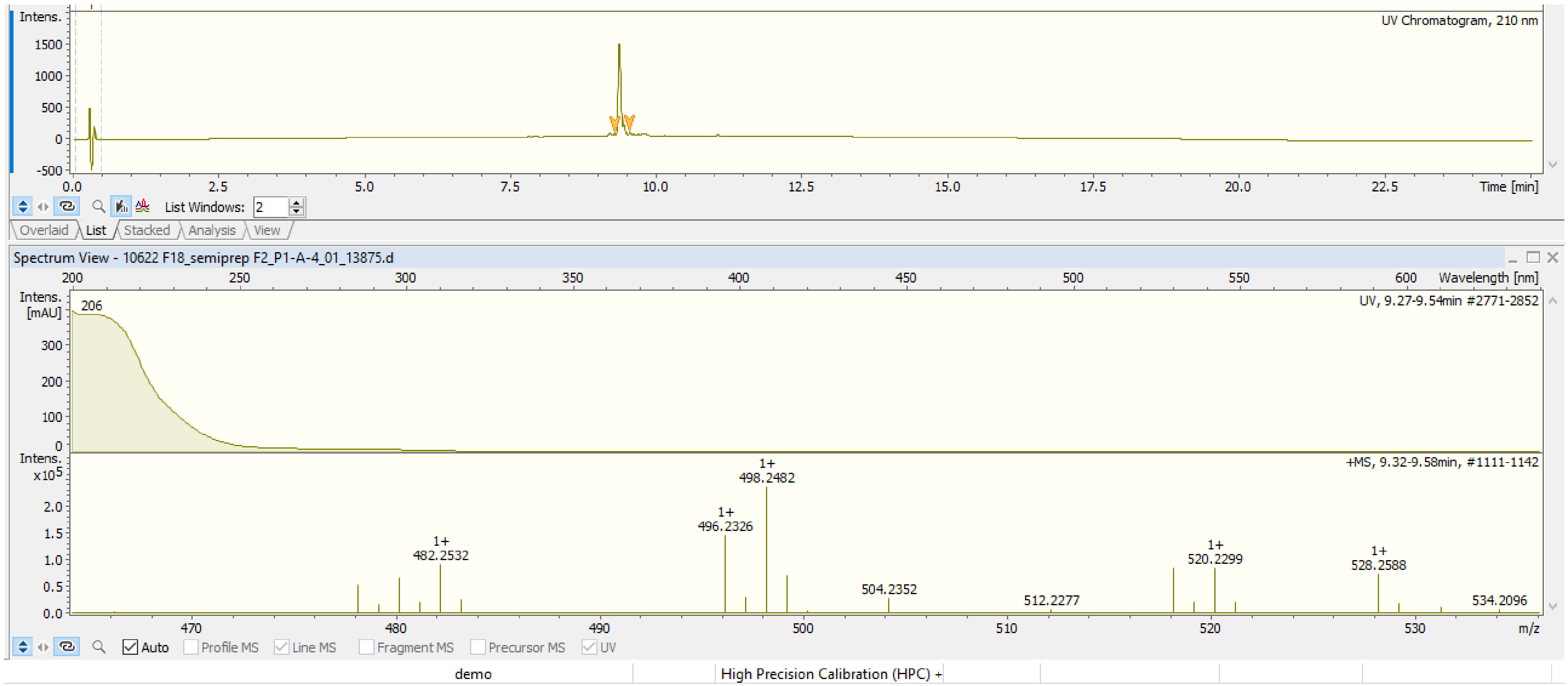

Figure S20: HR- ESI (+) MS data for cytochalasin K Steyn (**7**).

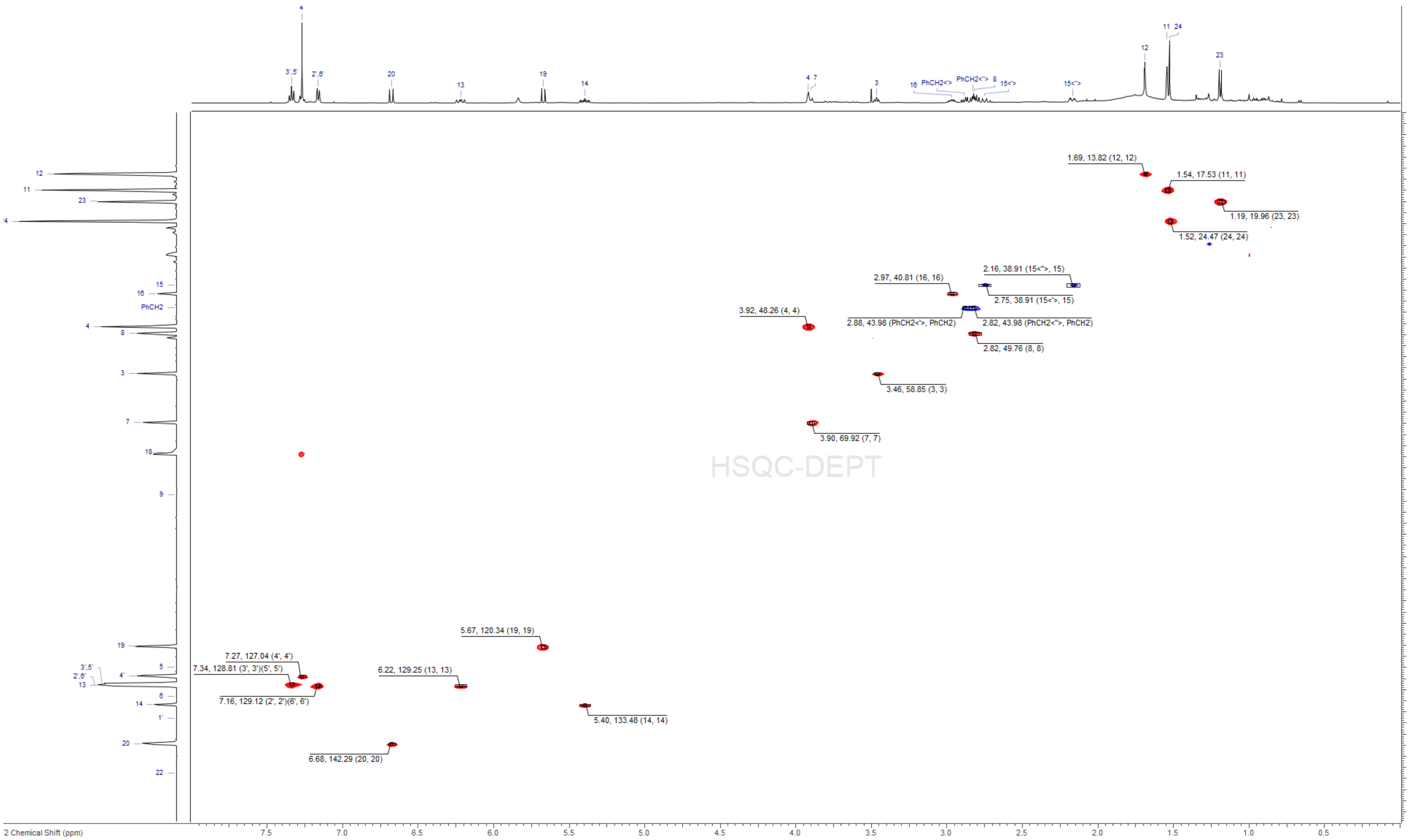

Figure S21. HSQC NMR spectrum (500 MHz, $CHCl_3$-*d*) of cytochalasin K Steyn (**7**).

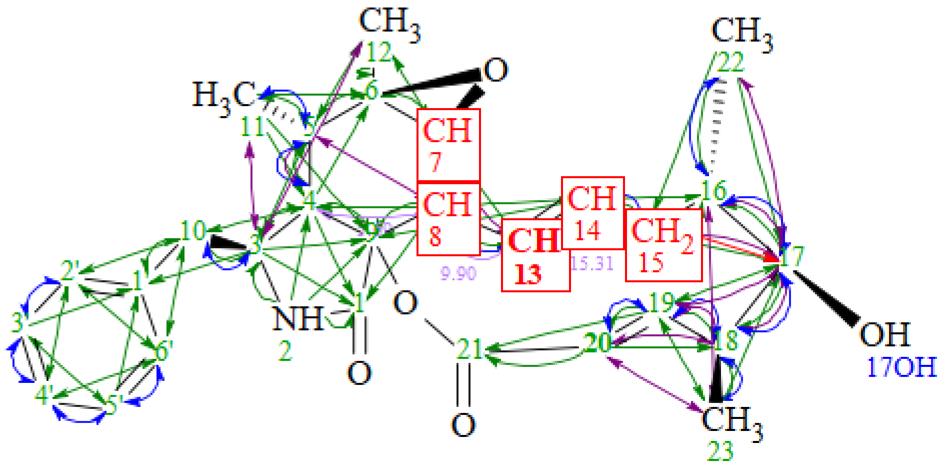

Figure 22. Key COSY (blue arrows), HMBC (green arrows) and ROESY (purple) correlations for **2**.

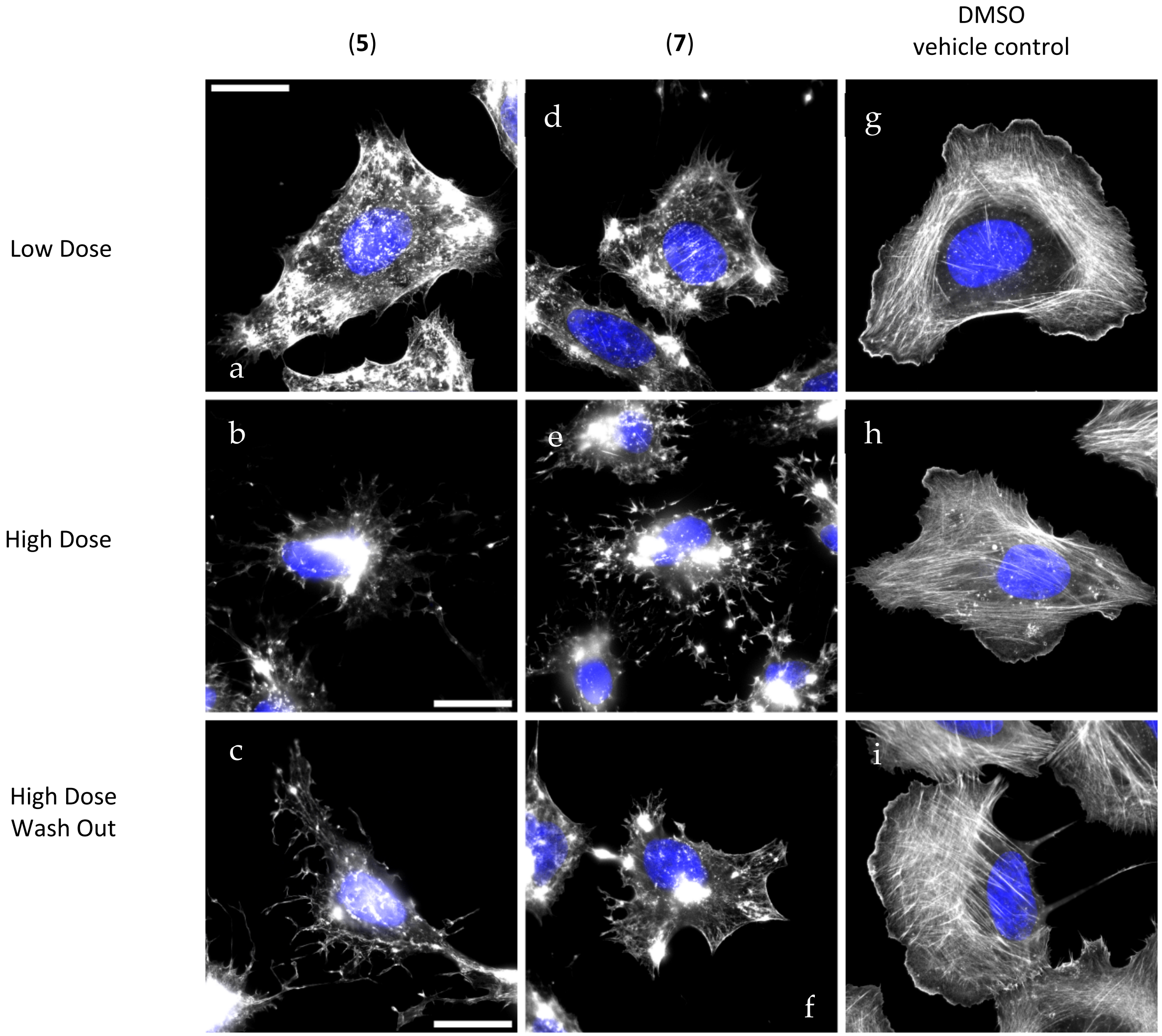

Figure S23: Overlay images of U-2OS cells depicting the effects of the cyclic carbonate cytochalasins **5** and **7**. Treatment concentrations were calculated based on previously determined IC_{50} values in mouse fibroblast cell line L929 (low dose: 1xIC_{50}; high dose 5xIC_{50}). Cells were fixed after 1h treatment using paraformaldehyde. Cellular filamentous actin (F-actin) and nuclear DNA were visulized using fluorecently-coupled phalloidin-ATTO488 (grey) and DAPI (pseudocolored in blue), respectively. The basic chemical structure of **5** and **7** differ only in the decoration of the six-membered ring, as **5** bears an epoxy group that is reduced to a double bond and hydroxyl group in **7** – shifting the ring into a more planar position. Both compounds induced severe effects, starting with the depletion of F-actin containing structures such as lamellipodia and stress fibres, and the formation of large actin accumulations after low dose treatment (Figure 2, a and b). The further increase of both compounds led to the complete collapse of the entire actin network (Figure 2, b and e), which was only partially reversible for **7** (Figure 2, f), and no reversible for **5** after wash out experiments (Figure 2, c).

Alignment of the sequences used in this study:

>DSM_116299_Nemania_diffusa_ITS_LSU

TACGCCAGCATCCTTGCAAAAGCGCGGTCCTCGGTCCCCGCAAGGGTATTATGCGGAGGGCTATAACACTCCCGAAAGAGCTACGTTCCCTGGGCTTTTATCCCCCTGCAGAAACCGATGCTGGCCTC
GACCTAGCGAAGTGCACCGGCGAAGAACACCGGATGATCCGCTAGGAAAAGGTCTGGTCGCAAACGCTTCCCTTTCAACAATTTCACGTGCTATTTAACCCTCTTTTCAAAGTGCTTTTCATCTTTCGA
TCACTCTACTTGTGCGCTATCGGTCTCTGGCCAATATTTAGCTTTAGAAGAAATTTACCTCCCATTTAGAGCAGCATTCCCAAACTACTCGACTCGTCGAAGGAGCTTTACAGAGGCTTGGTGTCCAAC
CGTACGGGGCTCTCACCCTCTAAGGCGTCCCGTTCCAGGGAACTCGGAAGGCACCGCGCCAAAAGCATCCTCTACAAATTACAACTCGGGCCCTAAAGAGCCAGATTTCAAATTTGAGCTGTTGCCG
CTTCACTCGCCGTTACTAGGGCAATCCCTGTTGGTTTCTTTTCCTCCGCTTATTGATATGCTTAAGTTCAGCGGGTATTCCTACCTGATTCGAGGTCAACCTTTTAGATACTGGGGCGTTTTACGGCAGG
GGACCGGTCCAACTATAGGCGAGGTGAGTAATTACTACGTCTAGAGTGTGAGCCGGCTCCGCCACTGACTTTGGGGAGCTACGGTGCCGTAGGCTCCCAACGCTAAGCAACAAGGGCTTAAGGGTT
GAAATGACGCTCGAACAGGCATGCCCACTAGAATACTAATGGGCGCAATGTGCGTTCAAAGATTCGATGATTCACTGAATTCTGCAATTCACATTACTTATCGCATTTCGCTGCGTTCTTCATCGATGC
CAGAACCAAGAGATCCGTTGTTGAAAGTTTTAACTTATTTAGTTATAGGTTCAGAATTCAACGATGAGACAGAGTTTCGTGGGCCACCGGCAGGGGTACCCGCGCCTTCCGCGTAGGAGCCTACGGG
GTAGGGCCTACGAGGGAGGCGCGACCTGCCGAGGCAACATGAGGTATGTTCACATGGGTTTGGGAGTTATTACACTCTTTAATGATCCCTCCGCTGGTTCACCAACGGAGACCTTGTTACGACTTTT
ACTTCCTCTAAATGACCAAGA

>DSM_116299_Nemania_diffusa_RPB2

GGGAACCCTACTTGCCAACTCTTTCGCAACATAGTTCGTCGGATGACTCAGGAAGTGCTATCTCACCTCAAGCGAAGTATCGAGCAAGGCAAGCAGTTCAATATTGCCCTTGCTGTCAAGTCAAACAT
CATCACGAGCGGACTGAAGTATTCTCTGGCCACGGGAAACTGGGGCGATCAGAAGAAGGCCATGAGCTCTACCGCTGGTGTTTCGCAGGTATTGAACCGATACACATTTGCATCCACCCTGTCACAT
TTGCGAAGAACGAATACACCGGTCGGCAGAGACGGCAAGCTTGCCAAGCCACGCCAGCTTCACAATACTCACTGGGGTCTCGTTTGCCCGGCCGAGACGCCCGAAGGTCAGGCTTGTGGCCTCGTC
AAAAACCTGTCCCTCATGTGCTCTATCAGCGTAGGTACTTCAACGGAACCAATTATCGAGTATATGATTTCTCGGAATATGGAAGTCCTAGAGGAGTACGAACCGCAAAGGTATCCTCACGCCACGAA
GATCTTTCTCAACGGATCATGGATCGGTGTCCATCAAGATCCAAAGGCTCTCGTCAAAGACGTCCAACGACTTCGTAGAACGAATCAGATTCCAGCTGAAGTATCCTTGATTCGGGAAATCCGTGACC
GTGAATTCAAGATCTTTTCAGATGCCGGCCGAGTCATGCGGCCTCTATACGTGGTCGAGCAGGATGATGACCCTGAGAACGGTATTAATAAGGGCACGTTGGTCTTAACGAAGAACATTGTGCGGC
GGCTTGAGATCGACCAAACTCTCCCACCTGGAAGTGAGGATTATTTTGGATGGCAAGGTCTAGTCAATGCCGGTGTCATCGAATATATGGACGCTGAAGAAGAGGAAACCGCCATGATCTGCATGA
CTCCCGAAGATCTAGAGGCTTATCGAATGGCCAAGGCAGGCATCGCTAACCCTGACGCGGAATATGACGTTAACAACCCGAACAAGCGACTAAAGACAAAAATAAACCCGACGACTCACACGTACA
CCCATTGCGAAATTCACCCAAGCATGCTTCTTGGCATTTGTGCCAGTATTATCCCGTTCCCCGATCATAACCAGGTAAGTACTATCGCCGTATATTTCGTAGCCGCTATTAATCATATTCTTAGTCACCCG
AA
